# Integrative RNA-seq and LASSO-COX analysis reveal Paeonol’s key target gene in proliferation suppression and apoptosis-induced in cervical cancer

**DOI:** 10.3389/fphar.2025.1646473

**Published:** 2025-10-07

**Authors:** Miao Li, Na Gao, Lei Xu, Peng Ge, Nan Jiang, Yuhong Shang

**Affiliations:** ^1^ Department of Obstetrics and Gynecology, The First Affiliated Hospital of Dalian Medical University, Dalian, China; ^2^ Department of General Surgery, The First Affiliated Hospital of Dalian Medical University, Dalian, China

**Keywords:** paeonol, cervical carcinoma, prognostic signature, differentially expressed genes, alternative splicing

## Abstract

**Background:**

The natural compound paeonol exhibits therapeutic promise against cervical carcinoma, though its precise molecular mechanisms remain undefined.

**Methods:**

First, we treated human cervical cancer (HeLa) cells with different concentrations of paeonol. Cellular proliferation and apoptotic responses were evaluated via cell-counting kit 8 (CCK8) assays and flow cytometric analysis. Subsequent transcriptomic profiling employed RNA sequencing coupled with alternative splicing assessment to detect differentially expressed genes (DEGs). Protein interaction networks were established for pivotal DEGs, followed by Gene Ontology (GO) and Kyoto Encyclopedia of Genes and Genomes (KEGG) pathway enrichment investigations. Clinical data pertinent to cervical cancer were retrieved from The Cancer Genome Atlas (TCGA). Prognostic model development incorporated Kaplan–Meier survival estimation, Least Absolute Shrinkage and Selection Operator (LASSO) regression, alongside univariate and multivariate COX proportional hazards analyses, with model accuracy subsequently assessed. Finally, Quantitative reverse transcription polymerase chain reaction (qRT-PCR) validated DEG expression.

**Results:**

Paeonol treatment suppressed proliferation while inducing apoptosis in HeLa cells. Transcriptomic and splicing analyses revealed 12 critical DEGs: NLRP1, FN1, NQO2, NREP, B4GALNT1, ANK3, FAM219A, ODF3B, MAPK15, EPGN, MUC1, and MEG3. Enrichment analyses indicated these DEGs principally associate with inflammatory processes and the biological regulation of cellular proliferation and apoptotic death. Analysis of clinical outcomes in 197 TCGA patients demonstrated significantly enhanced five-year survival probability within the low-risk cohort. FN1, NQO2, and ODF3B were incorporated into a prognostic signature following LASSO regression. Univariate and multivariate COX analyses identified T stage, tumor grade, and differential expression of these three genes as significant outcome predictors; the resultant prognostic model exhibited robust accuracy. qRT-PCR results corroborated the RNA sequencing data concerning DEG expression patterns.

**Conclusion:**

Paeonol modulates HeLa cell proliferation and apoptosis through regulation of 12 key genes, including FN1. This activity involves governing inflammatory responses alongside cellular proliferation, migration, and differentiation processes. These findings offer a theoretical foundation supporting paeonol’s potential clinical utility in cervical cancer management.

## Graphical Abstract

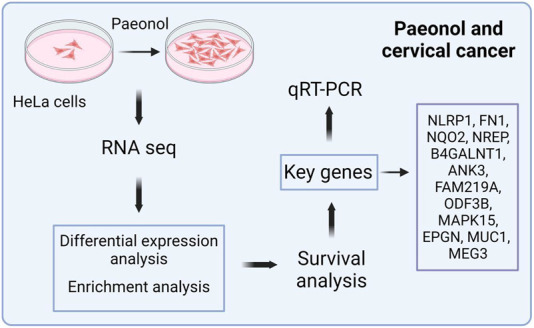

## 1 Introduction

Cervical carcinoma represents a prevalent malignancy affecting the female reproductive tract. Globally, its incidence constitutes the second most frequently diagnosed malignancy among women, surpassed only by breast cancer (Saei et al., 2018; Moore, 2006), and ranks fourth overall for female malignant neoplasms (Sharma et al., 2020). During 2020, approximately 604,127 new cervical cancer diagnoses occurred worldwide alongside 341,831 associated mortalities, reflecting an upward trajectory for both metrics (Singh et al., 2023). Within numerous developed nations, widespread implementation of Human Papillomavirus (HPV) vaccination programs coupled with routine screening has successfully diminished cervical cancer occurrence (Vu et al., 2018). Conversely, within many developing regions, incidence and mortality rates persistently escalate, necessitating novel therapeutic approaches. Current management strategies involve surgical intervention, radiotherapy, and chemotherapy, supplemented by second-line options including targeted agents and immunotherapies (Mauricio et al., 2021; Feng et al., 2020); these modalities, however, frequently incur adverse effects, substantial expense, and significant patient morbidity. Natural products have gained increasing attention in recent years as potential anti-neoplastic agents. Such compounds often offer advantages including multi-target activity, lower cost structures, and reduced adverse reaction profiles compared to conventional therapies (Naeem et al., 2022). Consequently, our investigation focuses on identifying novel natural product-derived therapeutics effective against this malignancy.

Alternative splicing (AS), or differential mRNA processing, denotes the mechanism generating varied mature transcript isoforms from precursor mRNAs. This process significantly expands proteomic diversity and functional complexity (Peng et al., 2022). Neoplastic cells frequently exhibit aberrant AS events yielding proteins that disrupt critical cellular functions—such as apoptotic regulation and cell cycle control—induce DNA damage, and consequently foster tumor initiation and progression. Furthermore, such aberrantly spliced isoforms can influence signaling pathways and drug targets, contributing to therapeutic resistance (Sciarrillo et al., 2020). Certain cancer-specific splicing variants possess utility as diagnostic biomarkers and potential therapeutic targets (Zhang et al., 2021). Cervical carcinogenesis may involve HPV-mediated dysregulation of splicing in normal cervical epithelia (Francies et al., 2021; Wu et al., 2018), motivating our search for agents capable of modulating pathological AS within cervical carcinoma cells.

Paeonol (2′-hydroxy-4′-methoxyacetophenone), a bioactive phenolic compound isolated from the dried root bark of Paeonia suffruticosa (Ranunculaceae family), demonstrates established anti-cancer properties. It suppresses malignant progression in breast, ovarian, and gastric carcinomas via mechanisms encompassing inhibition of neoplastic cell growth, induction of programmed cell death, cell cycle arrest, and modulation of diverse oncogenic signaling cascades (Sheng et al., 2021). *In vitro* studies confirm paeonol curbs proliferation, migration, and invasion capabilities of HeLa cervical cancer cells (Du et al., 2022), though its precise mechanistic underpinnings require elucidation; potential involvement of PI3K/AKT (Zhang et al., 2019), Nrf2/HO-1 (Hong et al., 2022), and JAK-STAT (Liu et al., 2023) signaling inhibition has been proposed. Additionally, paeonol reverses tumor cell resistance to chemotherapeutics like paclitaxel and cisplatin (Gao et al., 2019), enhances radiosensitivity, and synergistically suppresses HeLa cell proliferation when combined with cisplatin—likely through enhanced apoptotic induction (Han et al., 2016). Collectively, these findings underscore paeonol’s therapeutic promise for cervical cancer.

HeLa cells were treated with paeonol to evaluate its effects on cellular proliferation and apoptosis. Concurrently, transcriptomic profiling via high-throughput RNA sequencing (RNA-seq) was performed to discern potential molecular targets. Bioinformatics analyses were subsequently applied to cervical cancer clinical datasets sourced from The Cancer Genome Atlas (TCGA). Utilizing R software, survival analysis integrated these molecular signatures to establish a prognostic model, thereby evaluating paeonol’s translational potential. This integrated approach furnishes a molecular rationale supporting future clinical application of this compound.

## 2 Methods

### 2.1 Reagents

The following materials were utilized: Paeonol (Aladdin: H111081; 0.5 mg/mL), Dulbecco’s Modified Eagle Medium (Procell: PM150210), trypsin solution (Gibco: 25200072), fetal bovine serum (Hyclone: SH30084.03), and phosphate-buffered saline (PBS) (Life: C10010500BT). Proliferation assays employed RPMI-1640 medium (Hyclone, Inc.: SH30809.01B), fetal bovine serum (Gibco: 10099-141C), and a CCK-8 assay kit (Dongren Chemical: CK04).

### 2.2 Instruments

CO_2_ incubator (Heal force: HF90); Biological safety cabinet (Heal force: HF-1200LC); Inverted phase contrast microscope (Olympus: IX71); Electric constant temperature water bath (American standard: HH-US-A); Full-featured microplate detector (PerkinElmer/envision); Refrigerated centrifuge (Heal force: neofuge 15R).

### 2.3 Cell culture

HeLa cells were maintained in complete DMEM medium (Procell, Cat# PM150210) supplemented with 10% heat-inactivated fetal bovine serum (Hyclone, Cat# SH30084.03), 100 U/mL penicillin, and 100 U/mL streptomycin at 37 °C under 5% CO_2_ humidified atmosphere. For experiments, logarithmically growing cells were washed with PBS (Life Technologies, Cat# C10010500BT), detached using 0.25% trypsin-EDTA (Gibco, Cat# 25200072) for 1–3 min at 37 °C, and centrifuged at 1,000 rpm for 5 min. Cell density was determined via Trypan Blue exclusion using a 1:1 cell suspension/dye mixture. Remaining cells were pelleted (1,000 rpm, 5 min), resuspended, and seeded into 6-well plates at 4 × 10^5^ cells/well (n = 6 replicates per condition). Following overnight incubation, experimental groups received medium containing 0.5 mg/mL paeonol, while controls received paeonol-free medium. Incubation continued for 24 h prior to proliferation and apoptosis assessment.

### 2.4 Cell proliferation and apoptosis experiments

Cellular proliferation was quantified at 0, 24, 48, and 72 h post-treatment using CCK-8 reagent. Briefly, 10 μL CCK-8 solution was added per well, plates incubated (37 °C, 5% CO_2_, 4 h), mixed thoroughly, and absorbance measured at 450 nm.

Apoptosis was evaluated in 48 h-treated cells. Cells were collected, pelleted (1,000 rpm, 5 min), washed once with PBS, repelleted, and resuspended in 1× binding buffer. Annexin V-FITC (5 μL) and propidium iodide (10 μL) were added, followed by 15 min dark incubation at ambient temperature. Cells were diluted in 200 μL binding buffer and analyzed by flow cytometry. Comparative analysis employed Student’s t-test (significance threshold P < 0.05).

### 2.5 RNA-sequnce library construction

Total RNA (1 μg per sample; n = 6, 3 control, 3 treated) underwent purification with RNA clean beads and RQ1 DNase (Promega). RNA fragmentation and strand-specific library construction utilized the VAHTS Universal V8 RNA-seq Library Prep Kit for Illumina (NR605), involving sequential steps: first-strand synthesis, second-strand synthesis, end repair, adapter ligation, PCR amplification, and purification. Library concentration was determined via Qubit 4.0 fluorometry, and samples stored at −80 °C. Sequencing was performed on the Illumina Novaseq 6000 platform (PE150 mode) to generate high-quality transcriptomic data.

### 2.6 RNAseq analysis and differential gene expression analysis

Raw sequencing reads containing more than 2-N bases were first discarded. Subsequently, the raw reads were trimmed of adaptors and low-quality bases using a FASTX-Toolkit (v.0.0.13; http://hannonlab.cshl.edu/fastxtoolkit/). In addition, short reads of less than 16 nt were dropped to retain clean reads, which were subsequently aligned to the GRch38 genome by HISAT2. Uniquely mapped reads were ultimately used to calculate read number and paired-end fragments per kilobase of exon per million fragments mapped (FPKM) for each gene. The software DEseq2, which is specifically used to analyze the differential expression of genes, was applied to screen the RNA-seq data for DEGs. The results were analyzed based on the fold change (FC ≥ 2 or ≤0.5) and false discovery rate (FDR<0.05) to determine whether a gene was differentially expressed (Liu et al., 2022). Significantly upregulated and downregulated genes were visualized via heatmaps.

### 2.7 Alternative splicing analysis

RNA-seq data were interrogated to quantify alternative splicing events per sample. Differentially regulated alternative splicing events (RASE) in treated cells were defined (p-value ≤0.05) and associated with their corresponding genes (RASGs). Integration of RASG and DEG datasets identified key DEGs exhibiting concurrent differential expression and splicing alterations for subsequent investigation.

### 2.8 Enrichment analysis

Key DEGs underwent Gene Ontology (GO) and Kyoto Encyclopedia of Genes and Genomes (KEGG) pathway enrichment analyses using the “clusterProfiler” R package (significance P < 0.05), with results graphically represented. These analyses implicated paeonol in suppressing proliferation and inducing apoptosis in HeLa cells.

### 2.9 qRT-PCR

Quantitative reverse transcription polymerase chain reaction (qRT-PCR) was applied to validate RNA-seq-derived differential expression patterns of key DEGs. Quantitative PCR analysis was performed on RNA samples reverse-transcribed into cDNA using HiScript^®^ III RT SuperMix (+gDNA wiper) following genomic DNA elimination (42 °C, 2 min), with RT reactions conducted at 37 °C for 15 min and 85 °C for 5 s qPCR amplifications were carried out in 10 μL reactions containing 2 μL cDNA, 1 μL each of 10 μM gene-specific primers (GAPDH: F-GGTCGGAGTCAACGGATTTG/R-GGAAGATGGTGATGGGATTTC; NLRP1: F-GTCCCTCCTATTCCTCTTTG/R-GCCTAACAGCATCTCAGG; FN1: F-CCTCTTATCAACTGCATACT/R-GCATGATCTTGTTACTGTGA; NQO2: F-CATCCGAAGAAGAAAGAAAGG/R-CCTAGTGTGCTGCTTACG; NREP: F-AGGAGGGAGAGGAGTAATG/R-GTTGTTGTGTTAGCCAGTC; B4GALNT1: F-GAACAATGGACATCTACAAGG/R-CACTCTGCCTAATCTTCCTC; FAM219A: F-GGAGTGTTAAGGCAGTATCTA/R-TGGCAGCTTCTGTGTCTA; ODF3B: F-CTGGCTTCCGAGTGTTGT/R-GCCTATCAGGTCGTGAGT; MAPK15: F-ACGAACATGGATCTGAGGA/R-ACACCAGGAGTCGCCTAA; EPGN: F-GGAGTGGAGAGTTGAAGTT/R-AGGCAATCCTGTATTGTTTC; MUC1: F-TCTGAAGGAGGCTGTGAG/R-ACTTCTGCCAACTTGTAGG), and HieffTM qPCR SYBR^®^ Green Master Mix on a QuantStudio 12K system, using the following thermocycling protocol: 95 °C for 5 min; 40 cycles of 95 °C for 10 s and 60 °C for 30 s; followed by melt curve analysis (95 °C→60 °C→95 °C, 15 s/step). Gene expression was normalized to GAPDH and analyzed using the 2^−ΔΔCT^ method to compare untreated controls (n = 3) versus 0.5 mg/mL drug-treated cells (n = 3).

### 2.10 Survival analysis and construction and evaluation of pivotal gene prognostic model

Cervical cancer patient transcriptomic data, alongside clinical variables (age, disease stage), were retrieved from The Cancer Genome Atlas (TCGA; https://portal.gdc.cancer.gov/repository). Prognostically relevant genes and covariates were identified through Least Absolute Shrinkage and Selection Operator (LASSO) regression, followed by univariate and multivariate Cox proportional hazards analyses (Song and Shu, 2022; Xu et al., 2022). A predictive model was constructed, visualized via forest plot. Individual risk scores were computed [risk score = Σ (gene expression × regression coefficient)]. Patients were stratified into high-risk and low-risk cohorts based on median risk score, and survival differences assessed via Kaplan-Meier analysis using R (Wu et al., 2022). A nomogram integrated Cox regression results for prognostic visualization. Model calibration was evaluated using calibration curves.

### 2.11 Statistical analysis

Pairwise comparisons utilized Student’s t-test. Bioinformatics analyses employed R software. Survival outcomes were evaluated by Kaplan-Meier methodology. Prognostic determinants were identified via LASSO-Cox regression, enabling prognostic model construction.

## 3 Results

### 3.1 Impact of paeonol on cellular proliferation and apoptosis

**FIGURE 1:**
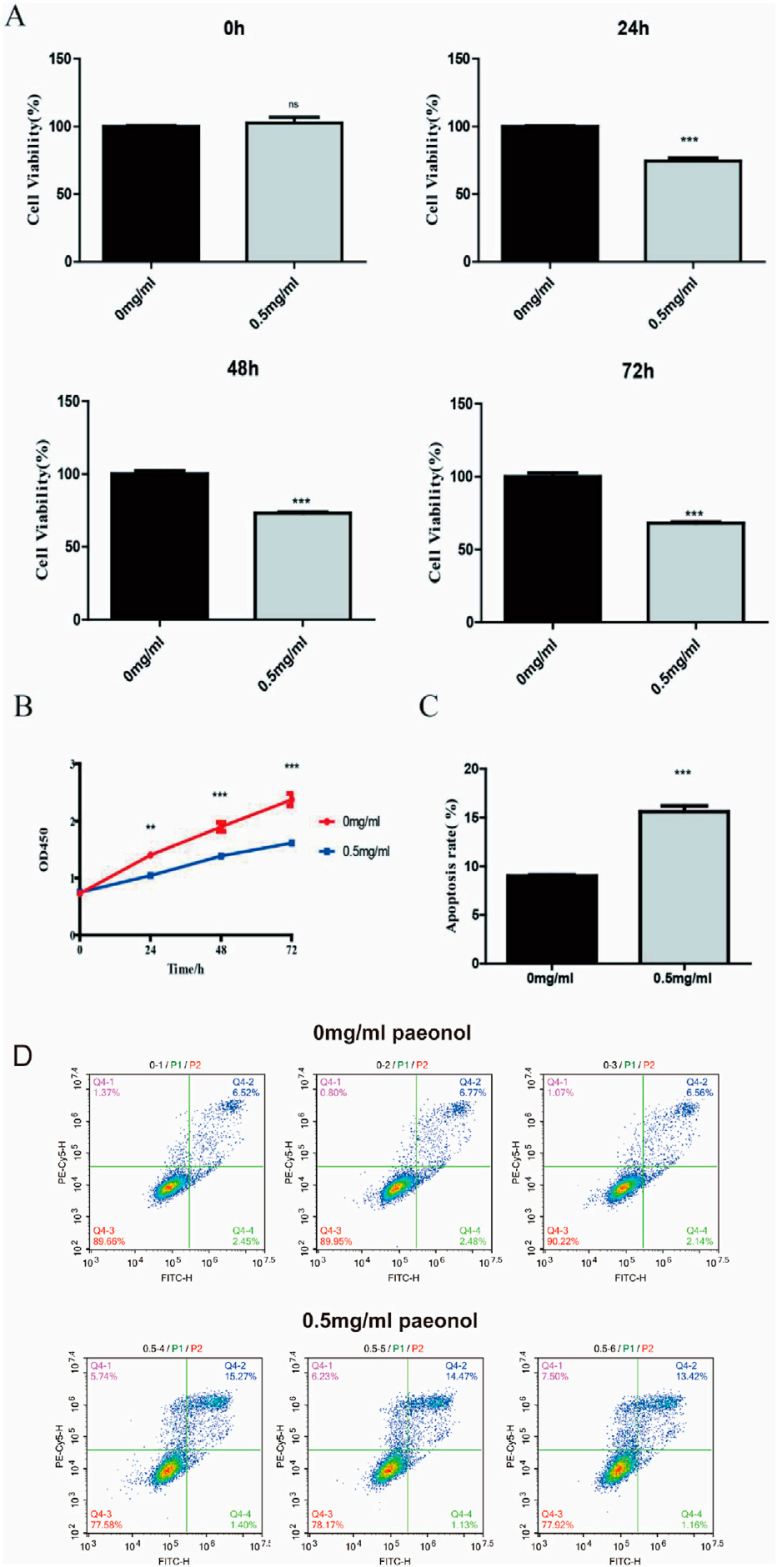
Impact of paeonol on HeLa cell proliferation and apoptosis. Effect of paeonol on cell proliferation **(A,B)** and apoptosis **(C)**. ** indicates that P value is less than 0.01. *** indicates that P value is less than 0.001. Flow cytometric analysis of cell apoptosis **(D)**, assessed by Annexin V-FITC/PI double staining.

Treatment with 0.5 mg/mL paeonol induced significant suppression of HeLa cell proliferation relative to untreated controls as observed effects in leukemia cell lines before (Kim et al., 2004). This inhibitory effect was statistically robust after 24 h (P = 0.0005) and persisted through 72 h (P = 0.0003) (Figures 1A,B). Furthermore, at this concentration, paeonol treatment markedly enhanced apoptotic cell death in HeLa cultures (Figures 1C,D).

### 3.2 Transcriptomic profiling outcomes

**FIGURE 2:**
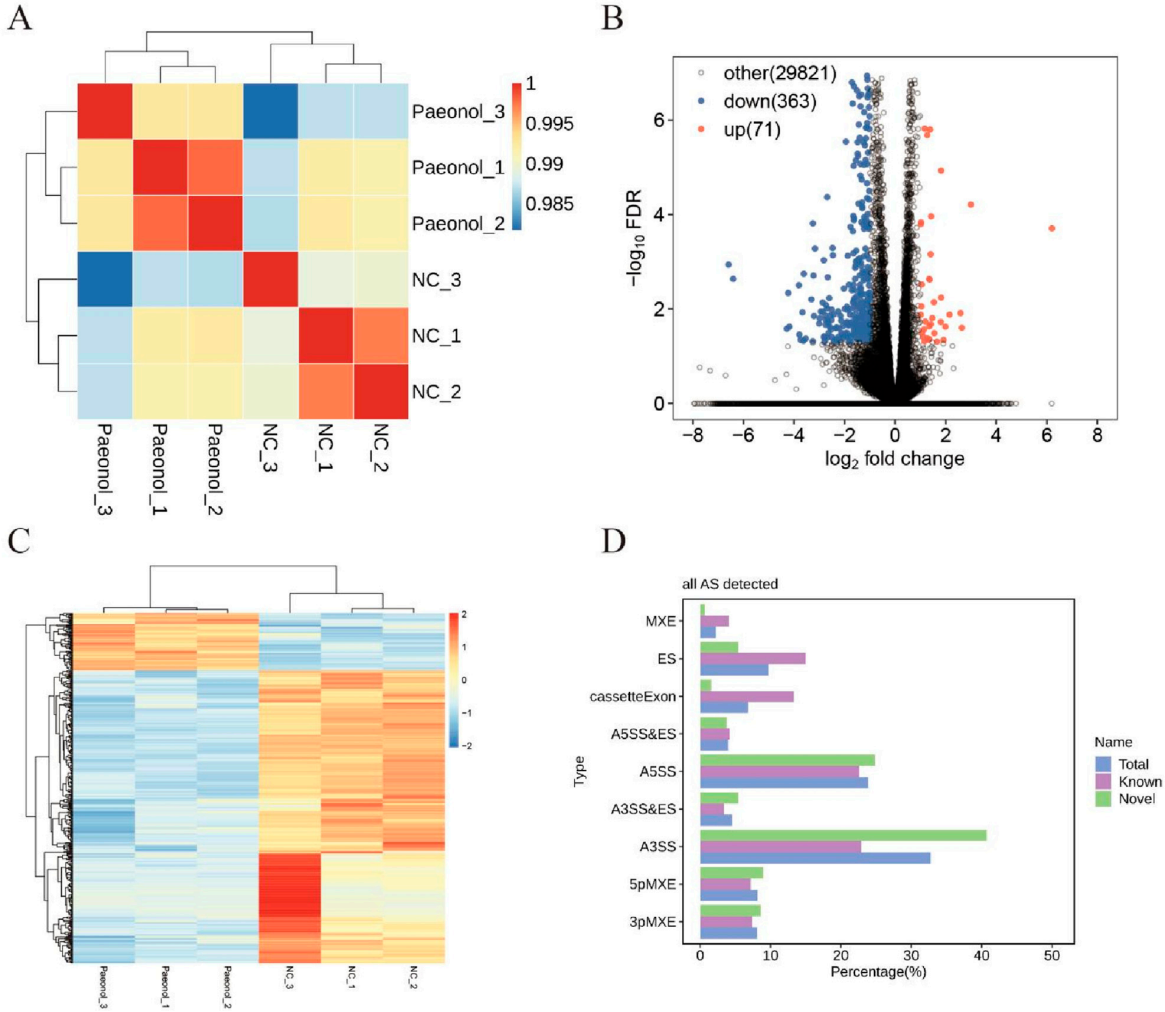
Transcriptomic Profiling Outcomes in Paeonol-Treated Cells. **(A)** Sample correlation clustering. **(B)** Differential expression volcano plot. **(C)** Per-sample gene expression heatmap. **(D)** Alternative splicing event detection.

High-quality sequencing reads (clean reads) were aligned against the HeLa cell reference genome (GRCh38, *Homo sapiens*), yielding uniquely mapped reads positioned exclusively at singular genomic loci. Distribution analysis of these uniquely mapped sequences across genomic regions revealed predominant enrichment within coding sequence regions (CDS), indicative of robust sequencing quality (Figure 2A). Consequently, uniquely mapped reads were retained for downstream analyses. Inter-sample gene expression correlation coefficients were computed, where elevated values denote substantial similarity in transcriptional profiles and data homogeneity. Conversely, diminished coefficients reflect pronounced differential expression or potential quality concerns. Proximity to unity signifies minimal inter-sample variation and fewer differentially expressed genes (Jazayeri et al., 2015). Cluster analysis demonstrated pairwise correlation coefficients exceeding 0.98 (Figure 2B), confirming high replicate consistency. These findings collectively indicate paeonol modulates a limited transcriptional repertoire (Zhou et al., 2022).

### 3.3 Differential gene expression

Comparative transcriptomics identified 434 differentially expressed genes (DEGs), comprising 71 significantly upregulated and 363 downregulated transcripts (Figure 2C). Hierarchical clustering of expression values revealed distinct transcriptional signatures between control and treated cohorts (Figure 2D).

### 3.4 Alternative splicing analysis

Nine splicing modalities were quantified: exon skipping (ES), alternative 5′ splice site (A5SS), alternative 3′ splice site (A3SS), mutually exclusive exons (MXE), mutually exclusive 5′ UTRs (5pMXE), mutually exclusive 3′ UTRs (3pMXE), cassette exon, A3SS&ES, and A5SS&ES. Among 105,078 detected splicing events, 46,568 corresponded to previously annotated isoforms while 58,510 represented novel events (Figure 2E).

Comparative analysis between experimental and control conditions identified 939 upregulated and 739 downregulated regulated alternative splicing events (RASEs). The A3SS and A5SS subtypes constituted the predominant fraction among all detected RASEs (Table 1).

**TABLE 1:** Distribution of differential variable splicing events (RASE).

Sample	Type	3pMXE	5pMXE	A3SS	A3SS&ES	A5SS	A5SS&ES	ES	MXE	cassetteExon	Total
Paeonol_vs._NC	Up	77	61	227	32	206	23	153	45	115	939
Paeonol_vs._NC	Down	62	56	176	20	163	27	88	43	104	739

Integrative examination of regulated alternative splicing genes (RASGs) and differentially expressed genes (DEGs) revealed twelve transcripts exhibiting concurrent marked transcriptional divergence and significant splicing alterations. These pivotal genes—designated key DEGs—comprise NLRP1, FN1, NQO2, NREP, B4GALNT1, ANK3, FAM219A, ODF3B, MAPK15, EPGN, MUC1, and MEG3. Transcriptional profiling demonstrated upregulation of NLRP1, B4GALNT1, FAM219A, NQO2, and EPGN, alongside downregulation of FN1, NREP, ANK3, ODF3B, MAPK15, MUC1, and MEG3 in treated cells (Table 2).

**TABLE 2:** Key DEGs.

Name	Description
NLRP1	NLR family pyrin domain containing 1
FN1	fibronectin 1
NQO2	N-ribosyldihydronicotinamide:quinone reductase 2
NREP	neuronal regeneration related protein
B4GALNT1	beta-1,4-N-acetyl-galactosaminyltransferase 1
ANK3	ankyrin 3
FAM219A	family with sequence similarity 219 member A
ODF3B	outer dense fiber of sperm tails 3B
MAPK15	mitogen-activated protein kinase 15
EPGN	epithelial mitogen
MUC1	mucin 1, cell surface associated
MEG3	maternally expressed 3

### 3.5 GO enrichment analysis and KEGG enrichment analysis of the key DEGs

**FIGURE 3:**
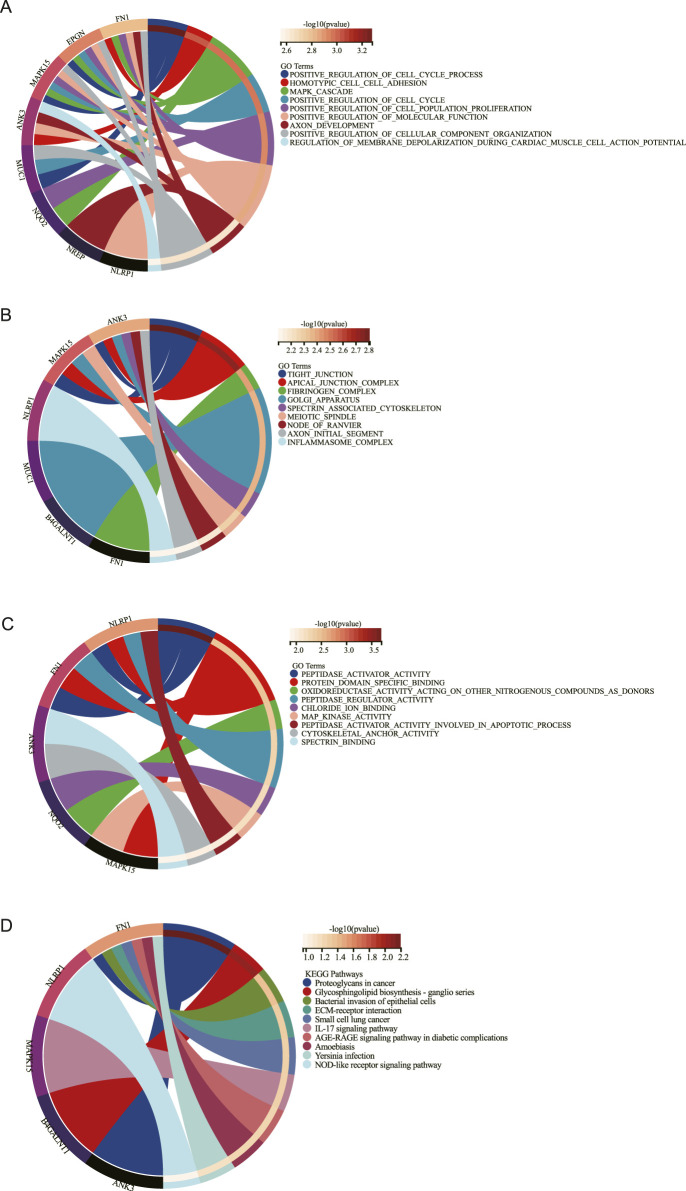
Functional enrichment analysis. GO enrichment of biology process (BP) **(A)**, cellular component (CC) **(B)**, and molecular function (MF) **(C)** enrichment analysis of DEGs. **(D)**. KEGG enrichment analysis of DEGs.

Results from GO and KEGG enrichment analyses of key DEGs are presented in Figure 3. The GO analysis identified enrichment in biological processes including positive regulation of cell cycle progression, MAPK cascade signaling, and positive modulation of cell population proliferation (Figure 3A). Molecular functions and cellular components implicated encompassed MAPK activity, protein domain-specific binding, and tight junction formation (Figures 3B,C). KEGG pathway analysis demonstrated significant enrichment for DEGs in: NOD-like receptor signaling, cancer-associated proteoglycans, ganglio-series glycosphingolipid biosynthesis, ECM-receptor interactions, and IL-17 signaling pathways (Figure 3D).

### 3.6 Survival analysis and prognostic model construction

**FIGURE 4:**
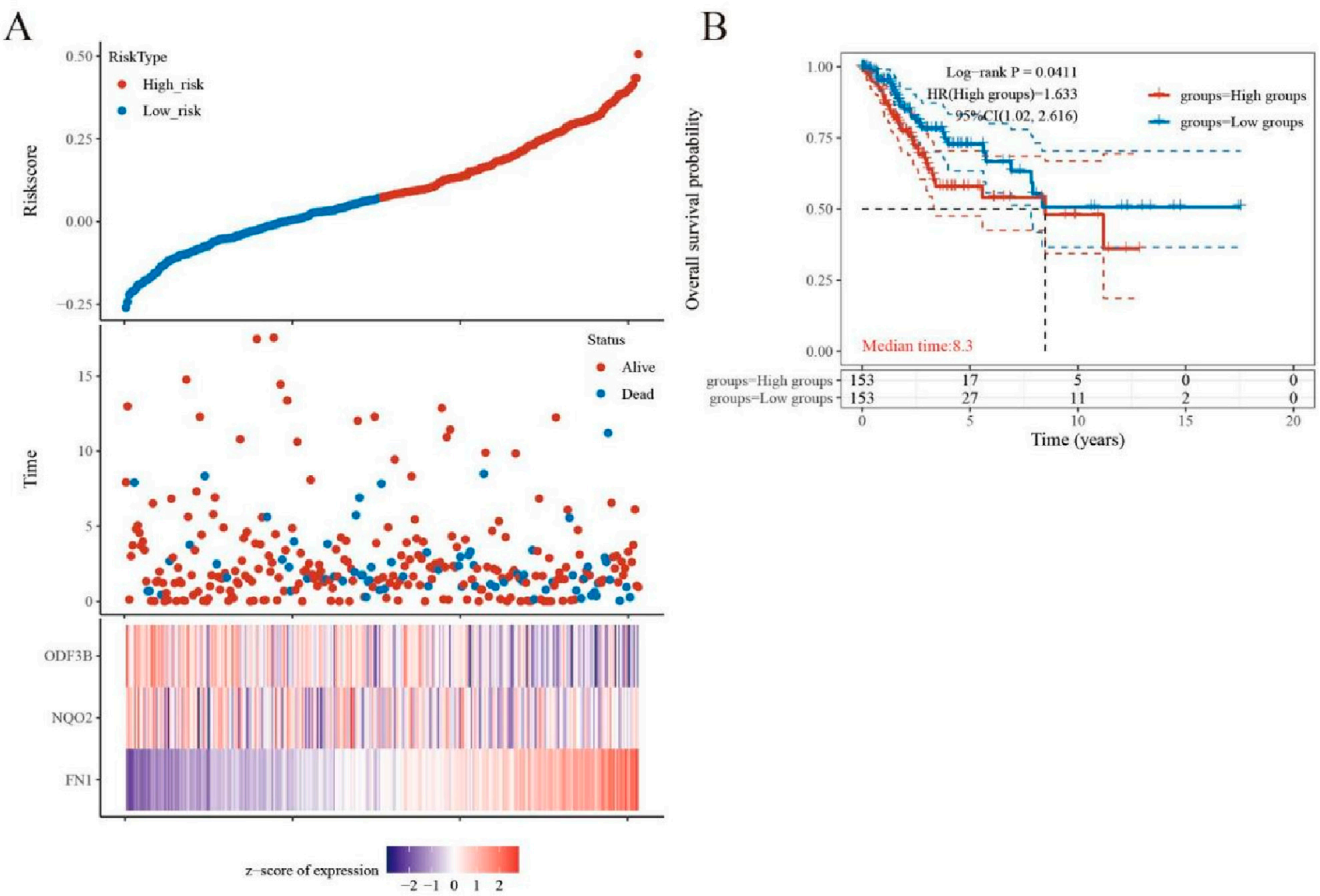
Diagnosis and survival prognosis analysis. **(A)** Grouping according to risk score and distribution of patient survival. **(B)** Kaplan-Meier survival anlysis.

Cervical cancer patients (n = 197) from TCGA were stratified into high-risk and low-risk cohorts based on analytical outcomes. Subsequent Kaplan–Meier analysis demonstrated significantly reduced five-year survival in high-risk versus low-risk patients (P = 0.0411; Figures 4A,B).

**FIGURE 5:**
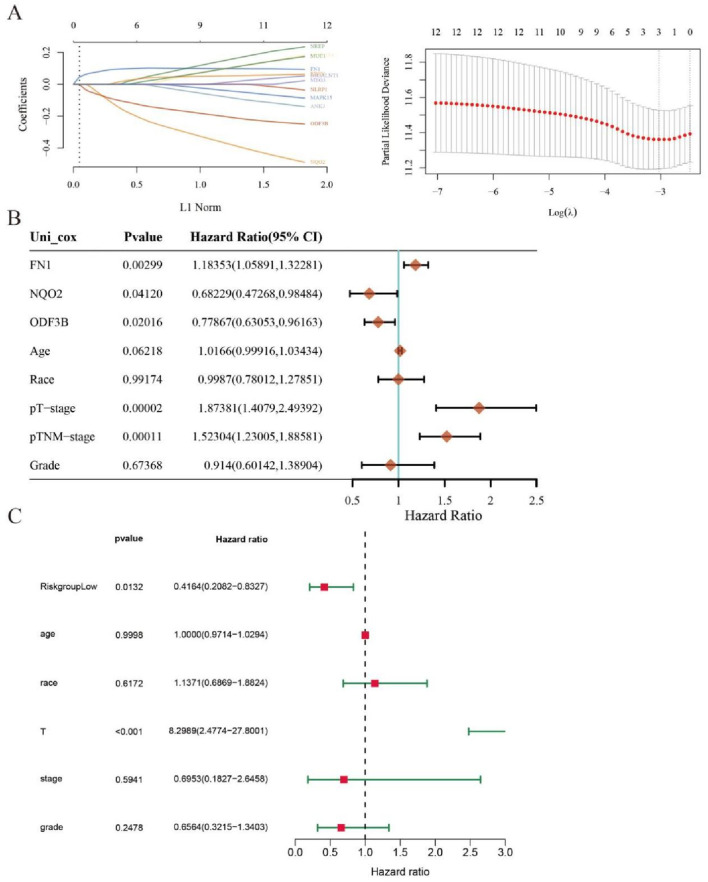
LASSO regression analysis. **(A)** LASSO regression analysis screens out genes for inclusion in the prognostic model. **(B)** Univariate COX analysis outcomes appear as a forest plot. **(C)** Forest plot for multivariate COX analysis.

Key DEGs underwent LASSO regression analysis to identify genes suitable for inclusion in prognostic models (Figure 5A). Three genes FN1, NQO2, and ODF3B, which log2foldchange is −1.67, 1.39, and −1.22, demonstrated significant associations with cervical cancer patient survival and were consequently incorporated into the prognostic model. Univariate COX regression indicated correlations between these genes and both tumor size (T stage) and TNM staging (Figure 5B), where T denotes primary tumor status, N represents lymph node involvement, and M indicates distant metastasis. Multivariate COX analysis (Figure 5C) revealed that tumor T stage and risk score exerted significant prognostic influence, with the risk score functioning as an independent outcome predictor.

**FIGURE 6:**
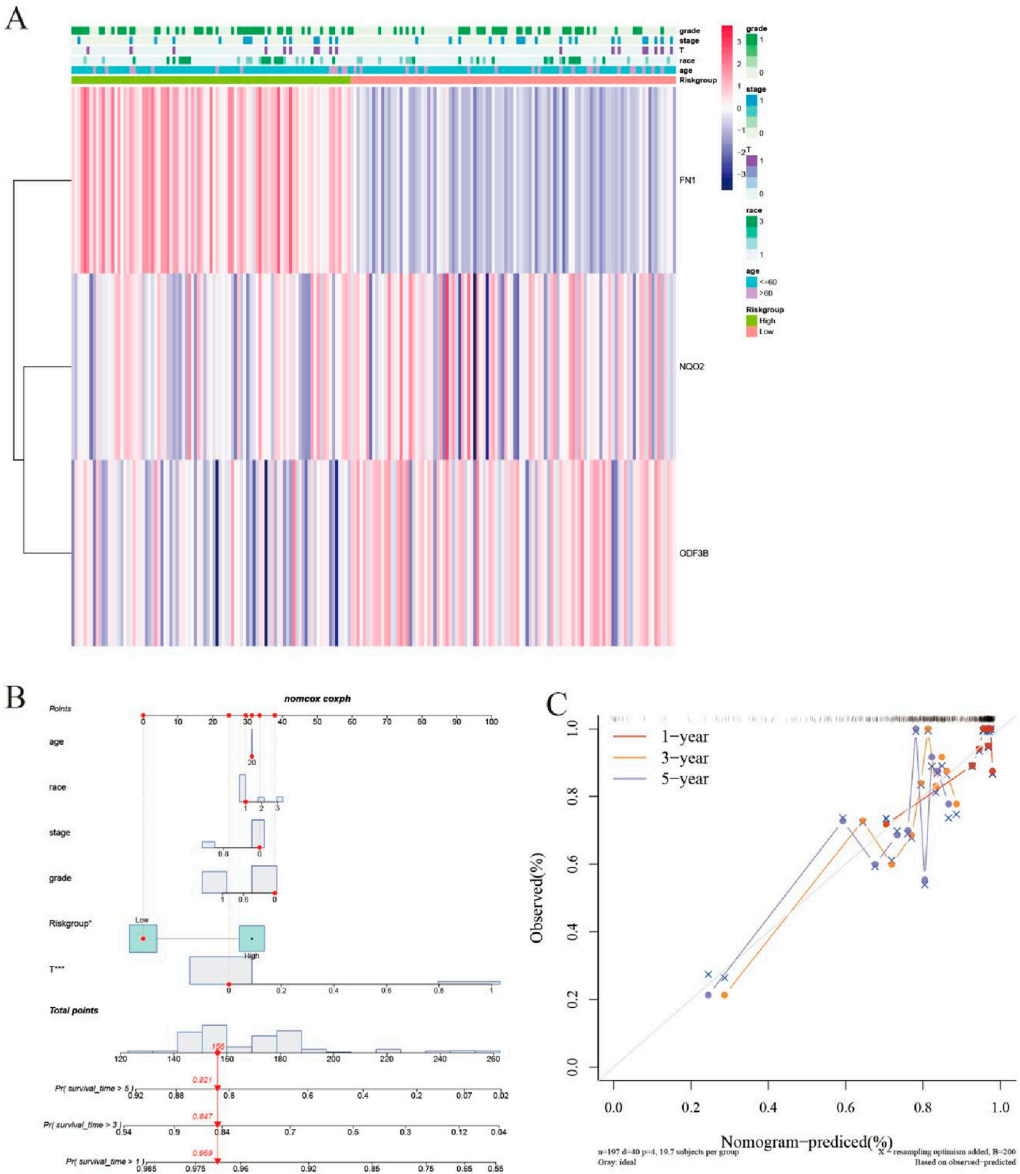
Evaluation of the efficacy of prognostic models. **(A)** Heat map. **(B)** Nomogram graph. **(C)** Calibration graph.

Correlating with improved survival outcomes, patients exhibiting lower risk scores were identified. A corresponding expression heatmap provides enhanced visualization of these findings (Figure 6A). Subsequent nomogram development based on the prognostic model (Figure 6B) enabled model validation via calibration curve assessment (Figure 6C).

### 3.7 qRT-PCR verification

**FIGURE 7:**
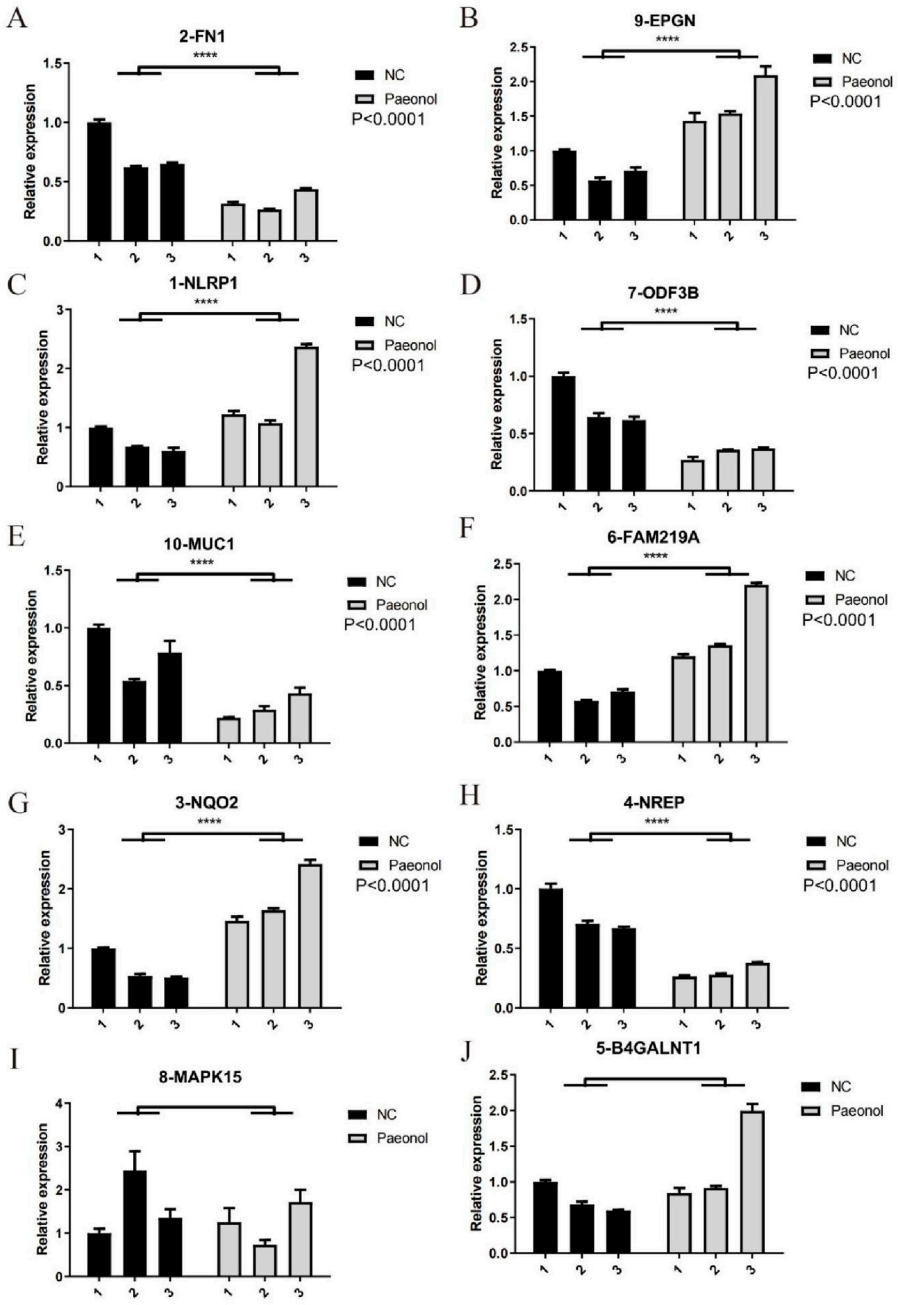
Expression levels of 10 key DEGs. **(A)** FN1, **(B)** EPGN, **(C)** NLRP-1, **(D)** ODF3B, **(E)** MUC1, **(F)** FAM219A, **(G)** NQ2, **(H)** NREP, **(I)** MAPK15, **(J)** B4GALNT1 quantified by qRT-PCR in Hela.

Quantitative RT-PCR measurements were performed for selected pivotal DEGs in experimental versus control cellular populations (Figure 7). Relative to controls, treated cells demonstrated elevated NLRP1, FAM219A, NQO2, and EPGN expression alongside reduced FN1, NREP, ODF3B, and MUC1 transcription levels.

## 4 Discussion

As a natural compound exhibiting anticancer properties, paeonol modulates serum glutathione peroxidase and superoxide dismutase activities in experimental models, suppressing lipid peroxidation and inflammatory responses to inhibit tumor growth (Sun et al., 2023; Morsy et al., 2022a). This positions paeonol as a potential therapeutic agent against cervical carcinoma. Our investigation assessed 0.5 mg/mL paeonol’s impact on HeLa cell proliferation and apoptosis, revealing significant proliferation suppression and apoptosis induction. Transcriptomic profiling identified 12 pivotal differentially expressed genes (DEGs)—NLRP1, FN1, NQO2, NREP, B4GALNT1, ANK3, FAM219A, ODF3B, MAPK15, EPGN, MUC1, and MEG3—potentially regulating cervical cancer cell behavior.

Functional enrichment studies implicated these DEGs in MAPK signaling cascades, cancer-associated proteoglycan pathways, extracellular matrix (ECM) recognition systems, NOD-like receptor signaling, IL-17 pathways, and metabolic processes. These molecular networks govern inflammatory cascades, cellular proliferation, migratory capacity, and apoptotic induction, collectively inhibiting neoplastic expansion.

Transcriptomic and qRT-PCR validation demonstrated elevated NLRP1, FAM219A, NQO2, and EPGN expression in treated cells. NLRP1, an inflammasome component, triggers pyroptosis during viral challenge (Morsy et al., 2022b), while NQO2 overexpression diminishes oxygen radical concentrations to restrict proliferation (Robinson et al., 2022). Conversely, FN1, NREP, ODF3B, and MUC1 exhibited reduced expression. FN1 facilitates cellular adhesion and motility (Lozinskaya et al., 2023; Wang et al., 2022; Cai et al., 2018), with elevated levels correlating with adverse clinical outcomes. NREP promotes tumor invasion and metastasis when overexpressed (Li et al., 2023; McDonough et al., 2005). MUC1 typically forms protective epithelial barriers (Kashyap and Kullaa, 2020), but its pathological overexpression in malignancies stimulates angiogenesis, proliferation, invasion, and chemoresistance while inhibiting apoptosis (Li et al., 2022; Chen et al., 2021; Nath and Mukherjee, 2014) through MAPK, Wnt, and PI3K/Akt pathway activation.

Thus, upregulated genes in paeonol-treated cells exert antitumor effects, while downregulated genes typically promote oncogenesis and treatment resistance.

MAPK cascades comprise three-tiered kinase modules MAPKKK (MAP3K), MAPKK (MKK/MEK/MAP2K), and MAPK (MK) regulating cellular growth, differentiation, inflammatory responses, and immune modulation (Widmann et al., 1999; Davis, 1993). These pathways influence numerous cancer-relevant processes (Gong et al., 2022), rendering components like JNK and p38 potential therapeutic targets (Dhillon et al., 2007). The ECM—comprising collagen, fibronectin, and glycoproteins (Nersisyan et al., 2021)—modulates cell survival, apoptosis, differentiation, and migration. ECM-receptor interactions critically influence tumor cell detachment, adhesion, proliferation, and death (Bao et al., 2019), with dysregulation implicated in gastric, prostate, colorectal, and glioblastoma pathogenesis (Andersen et al., 2018; Yan et al., 2018; Rahbari et al., 2016; Cui et al., 2018). As ECM constituents, proteoglycans facilitate tumor progression; certain HPV subtypes exploit proteoglycan receptors for cellular entry and transformation (Bartlett and Park, 2010).

NOD-like receptor signaling activates NF-κB, MAPK, and inflammasome pathways, driving IL-1β-mediated inflammation. While IL-17 normally confers protection, aberrant pathway activation stimulates ERK, JNK, and p38 cascades, upregulating IL-6, IL-1, and NF-κB to promote carcinogenesis (Amatya et al., 2017).

Enrichment analyses indicate pivotal DEGs modulate proinflammatory cytokine production, inflammatory regulation, and cellular proliferation/differentiation/migration/apoptosis. Systemic inflammation accelerates cancer progression through multifaceted mechanisms (Liang et al., 2022), highlighting inflammatory pathway regulation as crucial for oncogenesis control. Notably, downregulated DEGs associate with tumor-promoting pathways (IL-17, Wnt, ECM-receptor interactions), suggesting paeonol suppresses inflammatory and proliferative networks in HeLa cells.

Survival modeling revealed differential FN1, NQO2, and ODF3B expression between high-risk and low-risk cohorts. Elevated FN1 characterized high-risk patients, while NQO2 and ODF3B expression was diminished. Reduced FN1 with increased NQO2/ODF3B may inhibit inflammation and cellular proliferation/migration. Forest plots indicated hazard ratios >1 correlate with adverse outcomes. Calibration curves demonstrated model reliability when approaching ideal prediction lines. qRT-PCR validation confirmed RNA-seq differential expression patterns for key DEGs.

## 5 Conclusion

Integrating transcriptomic sequencing with bioinformatics approaches, this study establishes paeonol’s capacity to modulate critical DEGs including FN1 and MUC1. By governing inflammatory pathways and cellular proliferation/differentiation/invasion, paeonol inhibits cervical cancer cell growth while promoting apoptosis. These findings establish mechanistic foundations for paeonol’s clinical application in cervical cancer management and provide novel perspectives on natural product therapeutics. Further investigation must elucidate precise pharmacological characteristics to validate clinical translation potential.

## Data Availability

The RNAseq data presented in the study are deposited in the NCBI repository, available at: https://www.ncbi.nlm.nih.gov/geo/query/acc.cgi?acc=GSE305604.
